# Pulmonary vein isolation and beyond: Feasibility and acute outcomes of the lattice-tip dual-energy catheter for complex ablations

**DOI:** 10.1016/j.hroo.2025.11.013

**Published:** 2025-11-25

**Authors:** Ilaria My, Moritz Nies, Fabian Moser, Marc D. Lemoine, Laura Rottner, Katharina Govorov, Niklas Schenker, Alexander Zarfelder, Lauritz Schoof, Paulus Kirchhof, Bruno Reissmann, Feifan Ouyang, Andreas Metzner, Andreas Rillig

**Affiliations:** 1 Department of Cardiology, University Heart and Vascular Center Hamburg, University Medical Center Hamburg-Eppendorf, Hamburg, Germany; 2 German Center for Cardiovascular Sciences (DZHK), partner site Hamburg/Kiel/Lübeck, Germany

**Keywords:** Sphere-9, Linear lesion ablation, Atrial fibrillation, Pulmonary vein isolation, Thermal energy, Radiofrequency, Pulsed field ablation, Focal pulsed field, Lattice-tip catheter

## Abstract

**Background:**

A new large-footprint catheter that switches between pulsed field ablation (PFA) and radiofrequency ablation (RFA) and integrates with a novel 3-dimensional (3D) mapping platform has been introduced.

**Objective:**

This study aimed to evaluate the safety, efficacy, and procedural characteristics of the large-footprint dual-energy catheter in patients undergoing catheter ablation for atrial fibrillation or atrial tachycardia.

**Methods:**

Consecutive patients undergoing left (LA) and right atrial ablation for atrial fibrillation or atrial tachycardias were analyzed. All patients were enrolled in the prospective TRUST Registry (NCT05521451).

**Results:**

102 patients (37 women [36%]; median age 68 years [60–75]; median left ventricular ejection fraction 60% [53–60]) were included; 42 (41%) underwent first pulmonary vein isolation (PVI), and 57 (56%) repeat PVI. Median procedure and mapping times were 91 minutes (78–114) and 15 minutes (11.9–21.7), respectively; median 3D LA volume was 165 mL (142–199).

In 75 patients (42 index, 33 repeat procedures), pulmonary veins were targeted, and first-pass isolation was achieved in all using PFA only (median ablation 25 minutes [16–34]). Additional LA lines were applied in 87 of 102 (85%): anterior (34, 25 of 34 [73%] PFA + RFA), mitral isthmus (27, 18 of 27 [67%] PFA + RFA; 5 of 27 [18%] with PFA in the coronary sinus), posterior box (45, PFA only), and roof (23, PFA only). All lines were bidirectionally blocked, and posterior boxes isolated. A cavotricuspid isthmus line was created in 36 of 102 (35%; 30 of 36 [83%] RFA only). Procedural complications occurred in 3 of 102 (2.9%).

**Conclusion:**

The large-footprint dual-energy catheter enables safe and effective PVI, repeat PVI, and creation of complex left and right atrial lesion sets.

## Graphical abstract

**Figure:**
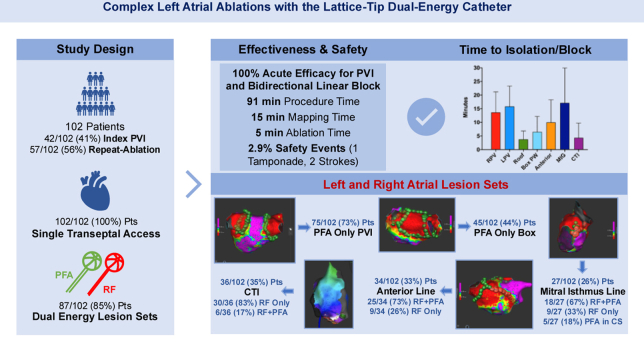

**Key Findings**

▪ This was a single-center, prospective study including 102 patients undergoing initial or repeat catheter ablation using a novel lattice-tip catheter capable of delivering both pulsed field and radiofrequency energy.
▪ The dual-energy lattice-tip catheter was feasible for complex left and right atrial ablations targeting multiple atrial substrates.
▪ Pulmonary vein isolation and linear lesions were achieved efficiently with 100% acute procedural success.
▪ Acute periprocedural safety events occurred in 3% of patients.
▪ Early remapping data demonstrated durable pulmonary vein isolation and persistent linear ablation sets, especially for mitral isthmus lines and posterior wall isolation.

## Introduction

In recent years, pulsed field ablation (PFA) has been established owing to its unique safety profile and efficacy for pulmonary vein isolation (PVI) procedures.^1^, ^2^, ^3^, ^4^, ^5^ Several ablation systems have been introduced into clinical practice but were mostly designed as single-shot devices for PVI.^1^^,^^6^^,^^7^ Therefore, data on ablation targets beyond PVI are limited.^4^^,^^8^, ^9^, ^10^, ^11^

The challenges for atrial ablation targets beyond PVI include (1) the variable architecture of the targeted myocardium with limited accessibility and/or catheter stability; (2) variable myocardial thickness, which can complicate lesion transmurality with conventional ablation modalities; and (3) anatomic proximity to vulnerable structures such as the phrenic nerve, the intracardiac conduction system, the esophagus, and the coronary arteries.^12^, ^13^, ^14^, ^15^, ^16^

The conformable 9 mm lattice-tip catheter with its large surface area allows for the creation of large and transmural lesions in the atria.^17^ It also facilitates catheter stability owing to its inherent friction.^18^ The ablation system’s ability to toggle between radiofrequency ablation (RFA) and PFA enables the operator to tailor ablation sets according to desired lesion characteristics and the surrounding anatomic structures at risk of damage.

These characteristics make the lattice-tip catheter highly promising to overcome current limitations for PVI and ablation lesions beyond. First preclinical and clinical data have been promising, but largely included patients undergoing their first atrial fibrillation (AF) ablation procedure with PVI as the dominant endpoint. Real-world data on complex atrial (re-)ablation procedures, particularly those performed under deep sedation, remain limited, given that previous studies of this ablation system have predominantly reported outcomes in patients undergoing their first left atrial (LA) ablation^4^^,^^9^^,^^10^^,^^19^, ^20^, ^21^ or, at most, 1 previous procedure.^11^

Preexisting fibrosis is also known to limit lesion formation for conventional RFA catheters. Therefore, it remains unclear whether the postablation scar compromises lesion formation or acute efficacy of the lattice-tip catheter.

This prospective, observational study reports on the ablation strategies, procedural safety, and acute efficacy of complex atrial ablation procedures using the lattice-tip catheter at a large tertiary care center. A detailed description of the methodology is provided, including energy selection, ablation time, acute outcomes, lesion durability, and repeat procedures in a subset of remapping cases.

## Methods

### Inclusion and exclusion criteria

Consecutive patients undergoing LA ablation procedures with the new system were enrolled in the prospective monocentric TRUST Registry at the University Heart and Vascular Centre Hamburg-Eppendorf, Hamburg, Germany (ClinicalTrials.gov ID: NCT05521451).

Exclusion criteria were any contraindications to oral anticoagulation, presence of intracardiac thrombi, pregnancy, severe mitral valve regurgitation, and more than mild mitral valve stenosis.

The research reported in this paper was conducted in accordance with the principles of the Declaration of Helsinki and approved by the institutional review board in compliance with regulations on human research. A written informed consent was obtained from all participants.

### Procedural setting

Initially, catheter ablation was conducted under general anesthesia. Thereafter, this was switched to deep sedation with midazolam, fentanyl, and propofol. 2 ultrasound-guided femoral venous accesses were obtained: one to advance a 7F steerable decapolar catheter (IBI, Abbott) into the coronary sinus (CS) and the second for a single transeptal puncture using a modified Brockenbrough technique and an SL1 sheath (8.5F, St. Jude Medical). Intravenous heparin was administered to maintain an activated clotting time (ACT) of ≥350 seconds. The catheter was only inserted into the vasculature after an ACT of ≥350 seconds had been achieved. Pulmonary vein (PV) ostia were tagged according to selective PV angiograms, local electrograms, and a 3-dimensional (3D) map.

### Ablation system

**Figure 1:**
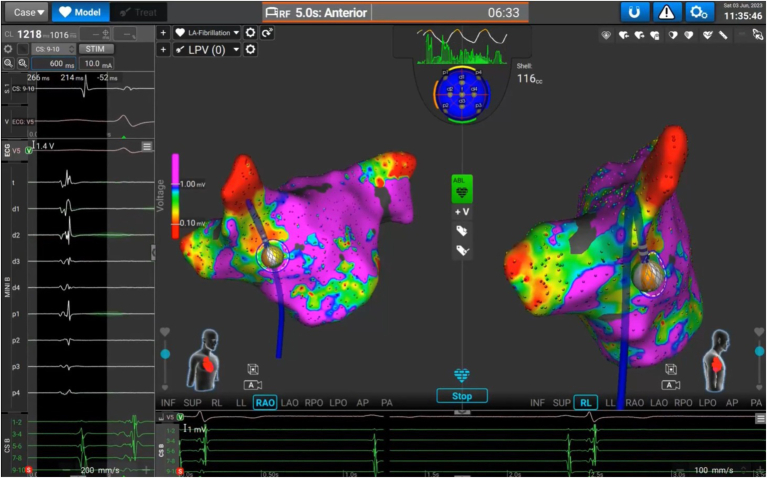
Catheter and mapping system. Sphere-9 catheter (Medtronic) visualized by the Affera mapping and ablation system.

The Affera mapping and ablation platform (Medtronic) has been described in detail before.^17^^,^^19^ 3D electroanatomic mapping is recorded via the 8F bidirectional Sphere-9 catheter (Medtronic) through a regular transeptal sheath SL1 without the need for sheath exchanges. Patients presenting in AF were cardioverted before mapping to allow a more precise evaluation of the LA substrate. The catheter features an expandable, irrigated 9 mm lattice tip equipped with 9 minielectrodes acting as thermocouples and mapping electrodes on its surface. A single indifferent electrode is centered inside the lattice tip, and 2 additional ring electrodes are located on the distal catheter shaft. An example of real-time mapping is presented in Figure 1.

The catheter is connected to proprietary generators designed for PFA (HexaPULSE, Medtronic) and RFA (HexaGEN, Medtronic), enabling the operator to toggle between PFA and RFA and tailor the ablation set to the patient’s or procedure’s demands. PFA is delivered using a biphasic monopolar waveform with an optimized energy setting (PULSE3), delivered over 4 seconds with saline irrigation^9^ and over 1 second for applications within the CS. RFA is applied under temperature control for 5 seconds, with saline irrigation, aiming for a surface temperature of 73°C–75°C. For both energy modalities, the target center-to-center distance between adjacent lesions is set at 5–6 mm for PFA and 8 mm for RFA. For the lateral PVs, a separate 3D map is created and merged with the LA map to avoid interpolation at the ridge between the PVs and the LA appendage (LAA), ensuring precise anatomic delineation. Ipsilateral PVs are circumferentially ablated using PFA solely. Even in the case of repeat PVI with electrical reconduction, complete circumferential reablation of the respective ipsilateral PVs is performed.

### Linear lesion sets

**Figure 2:**
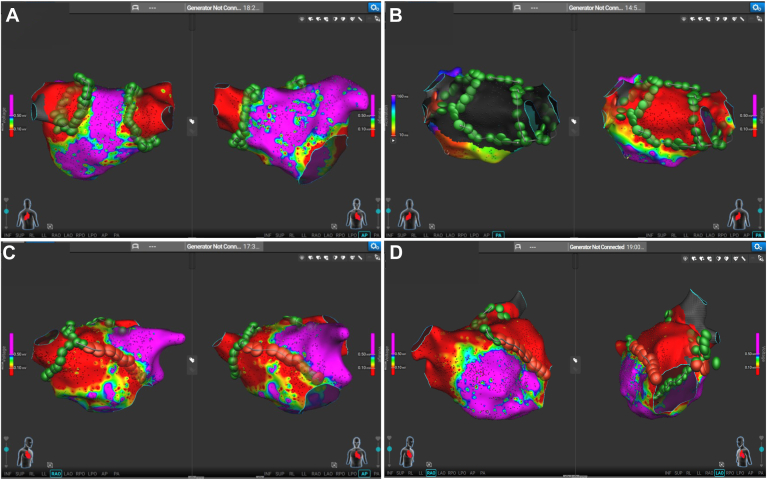
Representative cases of left atrial ablation sets. Bipolar 3-dimensional maps or activation map (panel B, left side) of exemplative cases. **A:** Circumferential pulmonary vein ablation. **B:** Posterior wall box isolation. **C:** Pulmonary vein isolation and anterior line ablation. **D:** Left atrial appendage isolation resulting from the anterior and mitral isthmus line. *Green tags* represent pulsed field ablation applications. *Red tags* indicate radiofrequency applications.

Additional linear lesion sets were created based on electroanatomic information as preplanned lines or to interrupt pathways of atrial tachycardias identified during the procedure. These lesions were created using either PFA or radiofrequency (RF) energy. For safety reasons, PFA was exclusively used for ablation of the posterior wall, including roof lines and posterior box lesions. Anterior wall lines, mitral isthmus lines, and focal ablations were performed using PFA, RFA, or a combination of both modalities. Representative cases are presented in Figure 2. Conventional pacing maneuvers and activation mapping were applied to prove the block of lines.

### Definition of complications

Major complications were defined as air embolism, pericardial tamponade, access site bleeding requiring intervention, phrenic nerve paralysis, transient ischemic attack, stroke, infection, myocardial infarction, atrioesophageal fistula, and death. Access site hematoma and pericardial effusion not requiring intervention were considered minor complications.

### Postablation care

Multiple transthoracic echocardiograms (at the end of the procedure, 2 hours after the procedure, and on day 1 after the procedure) were performed to rule out pericardial effusion. A figure-of-8 suture was applied to reach hemostasis. No protamine was routinely used. Oral anticoagulation was resumed 6 hours after the procedure.

### Statistical analysis

Data mean ± standard deviation was used to describe continuous variables with normal distribution; otherwise, median and interquartile range (first quartile, third quartile) were used. Nominal parameters are reported as absolute and relative frequencies. Data were summarized in an Excel sheet and analyzed with GraphPad Prism version 8.

## Results

### Baseline patient characteristics

A total of 102 patients were enrolled. Of these, 37 of 102 patients (36%) were female, the median age was 68 years (60–75), and the median body mass index was 27 kg/m^2^ (23-30). The median left ventricular ejection fraction was 60% (53–60), and the median CHA_2_DS_2_-VA score was 3 (1-4) (Table 1). 66 patients (65%) had persistent AF. The total LA volume, as calculated with the Prism-1 mapping software, was 165 mL (142–199), with a mean of 4158 ± 1470 recorded electrograms. 57 patients (56%) underwent repeat PVI, 42 patients (41%) index PVI, and 3 patients (3%) ablation for atrial tachycardia (all repeat ablation procedures). Among the repeat PVI group, 34 of 57 patients (59%) had previously undergone an RFA index procedure, 15 of 57 (26%) a cryo-PVI, 7 of 57 (12%) a PFA-PVI, and 1 of 57 (2%) a surgical RFA. Among the 60 patients (59%) with a history of previous ablation, 37 of 60 (62%) had undergone 1 previous procedure, 12 of 60 (20%) 2 previous procedures, and 11 of 60 (18%) more than 2 previous procedures. The presenting rhythm for the repeat ablation was sinus rhythm in 23 of 60 (57%), AF in 34 of 60 (57%), and atypical flutter in 3 of 60 (5%).

**Table 1:** Baseline patient characteristics

Baseline patient characteristics	N = 102
Female sex, n (%)	37/102 (36)
Age, y, median (Q1–Q3)	68 (60–75)
BMI, kg/m^2^, median (Q1–Q3)	27 (23–30)
LVEF, %, median (Q1–Q3)	60 (53–60)
CHA_2_DS_2_-VA, median (Q1–Q3)	3 (1–4)
Persistent AF, n (%)	66/102 (65)
First-do PVI, n (%)	42/102 (41)
Re-PVI, n (%)	57/102 (56)
AT, n (%)	3/102 (3)
Patients with previous ablations, n (%)	60/102 (59)
Patients with 1 previous ablation, n (%)	37/60 (62)
Patients with 2 previous ablations, n (%)	12/60 (20)
Patients with >2 previous ablations, n (%)	11/60 (18)

AF = atrial fibrillation; AT = atrial tachycardia; BMI = body mass index; LVEF = left ventricular ejection fraction; PVI = pulmonary vein isolation; Q1 = first quartile; Q3 = third quartile.

### Procedural characteristics and PV ablation results

Acute success of index PVI, repeat PVI, or linear or focal ablation was achieved in all 102 patients (100%). PFA alone was used for PVI, posterior wall ablation, and epicardial applications within the CS to minimize the risk of injury to adjacent structures such as coronary vessels, the phrenic nerve, and the esophagus.

The median procedure time was 91 minutes (78–114) including a mapping time of 15 minutes (11.9–21.7). The initial 17 of 102 patients (17%) were treated under general anesthesia owing to the putative risk of skeletal muscle capture with unipolar PFA leading to map shifts or extensive coughing. No significant map shifts occurred even when no muscle relaxants were used during anesthesia.^20^ The following 85 of 102 patients (83%) were treated in deep sedation only. In 75 patients, including 42 index PVI and 33 repeat procedures, the PVs were targeted, and first-pass isolation was achieved in all PVs using PFA only, except for 1 patient who required additional RF applications at the anterior left carina. Ablation of the lateral PVs was performed in 63 of 102 patients (62%), and ablation of the septal PVs in 75 of 102 patients (74%) (Table 2). The cumulative median ablation time for PVI was 25 minutes (16–34) overall, 14 minutes (11 -18) for lateral PVs, and 13 minutes (10-17) for septal PVs.

**Table 2:** Ablation data

Ablations performed	N = 102
Ablation of LPV, n (%)	63 (62)
Ablation of RPV, n (%)	75 (74)
Roof line, n (%)	23 (22)
Posterior wall box, n (%)	45 (44)
Anterior line, n (%)	34 (33)
Mitral isthmus line, n (%)	27 (26)
Applications in CS necessary, n (%)	6/27 (22)
CTI, n (%)	36 (35)
Lines or focal ablation in RA, n (%)	7 (7)

CS = coronary sinus; CTI = cavotricuspid isthmus; LPV = left pulmonary veins; RA = right atrium; RPV = right pulmonary veins.

### Linear ablation sets

In 87 of 102 patients (85%), additional LA linear lesion sets were performed (Table 2):

• Anterior lines (connecting the mitral annulus with the right superior PV) were created in 34 of 102 patients (33%). All lines were bidirectionally blocked at the end of the procedure. Of these, 9 of 34 (26%) were created using RF energy only, and 25 of 34 (73%) using a combination of PFA and RF. The median ablation time was 6 minutes (4-12).
• Mitral isthmus lines were performed in 27 of 102 patients (26%), and acute bidirectional block was achieved in all cases. RF only was used in 9 of 27 patients (33%), whereas PFA + RF was used in 18 of 27 patients (67%). In 5 of 27 patients (18%), additional PFA applications were delivered within the CS. The median ablation time was 12 minutes (6-25).
• Posterior box lesions were completed in 45 of 102 patients (44%) using PFA only, achieving acute posterior wall isolation in all cases. The median ablation time was 5 minutes (4-8).
• Roof lines were performed in 23 of 102 patients (23%), all of which were successfully blocked using PFA only. The median ablation time was 3 minutes (2-4).
• Cavotricuspid isthmus ablation was performed in 36 of 102 patients (35%), all with acute bidirectional block. RF only was applied in 30 of 36 patients (83%), whereas a combination of RF and PFA was used in the remaining 6 of 36 patients (17%). The median ablation time was 2 minutes (2-4).
• Right atrial ablation (focal or linear RF applications) was performed in 7 of 102 patients (7%).

**Figure 3:**
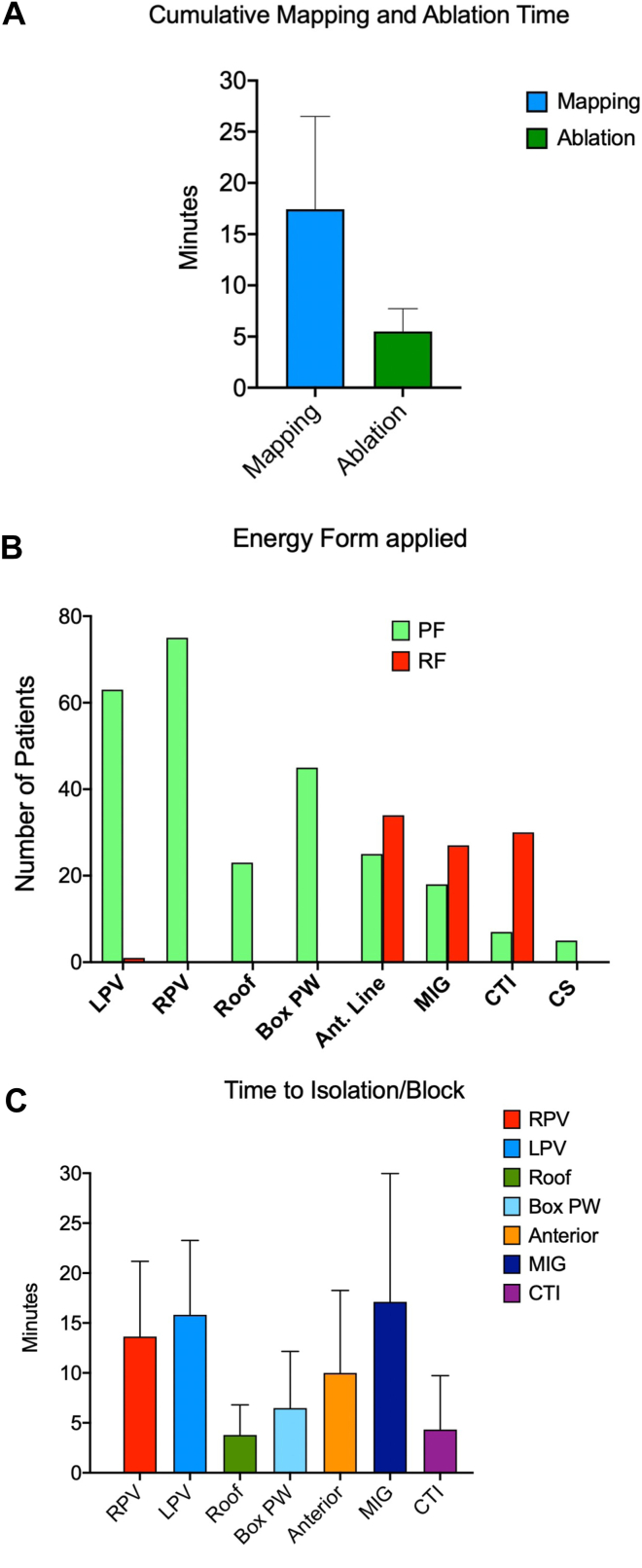
Ablation data. **A:** Mapping and ablation time per patient. **B:** Quantification of the energy form applied. **C:** Time to isolation or block per lesion set. Ant. = anterior; CS = coronary sinus; CTI = cavotricuspid isthmus; LPV = left pulmonary veins; MIG = mitral isthmus; PAC = premature atrial contraction; PF = pulsed field; PW = posterior wall; RF = radiofrequency; RPV = right pulmonary vein.

In 4 of 102 patients (3.9%), a subsequent LAA isolation was performed. The cumulative ablation time per patient was 5.3 minutes (4-7), with a median of 78 lesions (56–106) applied (see Table 3 and Figure 3A). The types of energy used and ablation durations for each lesion set are summarized in Figure 3B and 3C.

**Table 3:** Procedural characteristics

Procedural characteristics (median, Q1–Q3)	N = 102
Procedure time (min)	91.5 (78–113.75)
Mapping time (min)	15 (11.9–21-7)
Ablation time (min)	5.3 (3.8–7)
Ablation lesions (n)	78 (56–106)
LA volume, 3D mapping (mL)	165 (142–199)

3D = 3-dimensional; LA = left atrial; Q1 = first quartile; Q3 = third quartile.

### Safety

Acute periprocedural safety events were recorded in 3 of 102 patients (3%) (Table 4). Cardiac tamponade requiring pericardiocentesis occurred in a 70-year-old male with a body mass index of 27 kg/m^2^. Tamponade was successfully treated with pericardiocentesis via a subxiphoid approach. The tamponade was detected in transthoracic echocardiography at the end of an otherwise uneventful procedure, during which only the PVs were ablated using PFA. A blood gas analysis of the effusion showed PCO_2_, PO_2_, and pH values suggesting a venous origin.

**Table 4:** Safety events

Safety events	N = 102
Cardiac tamponade, n (%)	1/102 (1)
Vascular complications, n (%)	0/102 (0)
TIA or stroke, n (%)	2/102 (2)
Bleeding, n (%)	0/102 (0)
Myocardial infarction, n (%)	0/102 (0)
Phrenic nerve paralysis, n (%)	0/102 (0)
Atrioesophageal fistula, n (%)	0/102 (0)
Death, n (%)	0/102 (0)

TIA = transient ischemic attack.

One 79-year-old female with a CHA_2_DS_2_-VA score of 6 presented with transient dysarthria, diplopia, and ataxia 1 hour after an otherwise uneventful procedure. Immediate computed tomography revealed no intracranial hemorrhage or infarct demarcation. However, magnetic resonance imaging (MRI) on the next day showed small, subacute left-sided mesencephalic and hemispheric infarctions. Given that subsequent neurovascular evaluation revealed no abnormal findings, periinterventional embolism was suspected. The patient had only a partial recovery 6 months after the stroke. During the procedure, ACT had been >350 seconds, and no catheter or sheath exchanges had been performed. No charring, thrombi, or other abnormalities had been noted on the lattice tip upon catheter removal.

Another 54-year-old male (CHA_2_DS_2_-VA score 2) reported postinterventional diplopia. Again, cranial computed tomography did not reveal any signs of acute embolism. However, MRI revealed chronic and subacute cortical microinfarctions but without a clear correlation to the acute symptoms. The high-risk patient (terminal renal failure with hemodialysis, history of mitral valve endocarditis, severe coronary artery disease) had several competing causes for embolism. Chronic brain MRI lesions clearly indicate that other sources of embolism must have contributed to the MRI findings, but periinterventional microembolism could not be ruled out. The patient was admitted to the stroke unit, but the clinical symptoms quickly subsided (National Institutes of Health Stroke Scale score 0) and the patient recovered completely. It remains unclear whether the brain lesions were associated with the procedure and periinterventional neurologic symptoms or merely coincidental.

There were no other procedure-related serious adverse events. No patient reported acute symptoms attributable to esophageal affection or injury, and no ST-segment changes suggesting coronary artery spasm were observed. No patient presented intraprocedural phrenic nerve palsy. Minor complications were not observed (Table 4).

### Durability and repeat procedures

During a median follow-up time of 102 days (39–187), a total of 11 of 102 patients (11%) underwent repeat procedures. Of the 7 patients who underwent repeat ablation owing to arrhythmia recurrence after PVI in the previous procedure, 6 of 7 (86%) demonstrated durable PV isolation and only 1 patient (14%) exhibited reconduction of the lateral PVs, at a mean follow-up of 4 months after the index procedure. 3 of 11 patients (27%) had previously received posterior wall isolation; all 3 (100%) maintained durable isolation at the time of the repeat procedure. In contrast, 5 of 11 patients (45%) had undergone anterior line ablation during the initial procedure, and in 4 of 5 (80%), reconduction was observed during the repeat. 4 patients (36%) underwent planned implantation of LAA-occlusion devices 5–8 weeks after electrical isolation of the LAA. Durable LAA isolation was confirmed in 2 of 4 patients (50%), whereas reconduction was observed in the remaining 2 patients (50%), in both cases owing to anterior line reconnection (1 anterior line previously done with RF only, 1 with RFA/PFA). Mitral isthmus lines were durably blocked in all 3 of 3 patients (100%). Data from remap procedures are presented in Table 5, and detailed information on arrhythmia recurrence is presented in the Supplemental Material.

**Table 5:** Repeat procedures

Patient	# previous ablation	Previous lesion set	Previous energy sources	Affera lesion set	Findings at repeat ablation
**1**	0	n/a	n/a	- PVI - Roof line	- PVI + roof line durable - Reablation for PACs
**2**	0	n/a	n/a	PVI	PVI durable
**3**	0	n/a	n/a	- PVI - Anterior line - CTI	- PVI durable - Anterior line reconnected
**4**	0	n/a	n/a	- PVI - Roof line	- PVI+ roof line durable
**5**	0	n/a	n/a	- PVI - Anterior line - Roof line	- PVI + roof line durable - Anterior line not blocked
**6**	0	n/a	n/a	PVI	- Left PVs reconnected
**7**	2	- PVI - Re-PVI (all PVs)	- Cryo-PVI - Redo with RFA	- Posterior box - Anterior line - CTI	- Posterior box + CTI durable - Anterior line reconnected - Reablation for AF + AT
**8**	1	PVI	- Farapulse	- Posterior box - Anterior line - Mitral isthmus	- Box+ mitral isthmus durable - Anterior line reconnected
**9**	4	- PVI - Re-PVI - Posterior box - Anterior line + mitral line (not blocked)	- Cryo-PVI - Redo RFA	- Re-posterior box - Re-anterior line - Re-isthmus line - CTI	All durable
**10**	0	n/a	n/a	PVI + roof line	All durable
**11**	5	- CTI 05 - PVI 2014 - Re-PVI + re-CTI 2015 - Surgical PVI 2022 - Re-PVI 2023	- First-do unclear - Surgical, RF	- RA lines (anterior + posterolateral) - Mitral line - Focal anterior wall ablation - Re-re-CTI	- CTI durable - LA lesion not assessed (reablation right atrial only)

AF = atrial fibrillation; AT = atrial tachycardia; CTI = cavotricuspid isthmus; LA = left atrium; n/a = not available; PVI = pulmonary vein isolation; RA = right atrium; RF = radiofrequency; RFA = radiofrequency ablation.

## Discussion

The present prospective analysis of 102 patients undergoing LA ablation with a novel lattice-tip device capable of delivering PF and RF energy demonstrates the following:

1) The feasibility of the novel system is confirmed in patients undergoing repeat procedures (59% of the present cohort) targeting different LA substrates.
2) Linear lesions can be delivered rapidly with high acute success.
3) Initial data from a small number of remapping studies show convincing durability of PVI (all PVs isolated in 6 of 7 patients) and decent durability for linear ablation sets, especially for mitral isthmus lines and posterior wall isolation (100% durable block/isolation, respectively).

### Patient and procedural characteristics

We report on 1 of the largest patient cohorts treated with the lattice-tip ablation system thus far. In total, 59% of procedures were reablation procedures, and more than 30% of patients had undergone more than 1 previous procedure. The previous reports on this ablation system predominantly reported outcomes in patients undergoing a first LA ablation procedure^4^^,^^9^^,^^10^^,^^19^^,^^21^ or a maximum of 1 previous procedure.^11^ Two features of the new system, the ability to rapidly acquire precise anatomic and electrical information in the LA and the ability to choose the ablation energy, suggest particular usefulness in patients with previous ablations. Complex substrates owing to atrial cardiomyopathy and previous ablation lesions require accurate, extensive mapping. This likely explains the slightly longer mapping times noted in this study than previous publications.^19^^,^^22^ The present mapping times are comparable with mapping times in 1 other study reporting on reablations with the lattice system.^11^ Interestingly, total procedure time remained comparable with previous reports, which highlights the suitability and efficacy of the ablation system for complex atrial procedures.

Most procedures in this study were performed under deep sedation. This is important to note given that general anesthesia is not readily available in a number of health care systems, often owing to cost constraints or limited availability of anesthesiologists for cardiac ablation procedures. General anesthesia was only performed during the initial phase of clinical experience with the ablation platform owing to concerns about extensive skeletal muscle capture leading to map shifts or coughing. However, as previously reported,^8^^,^^20^ no differences in acute effectiveness or safety and no map shifts were observed.

### PVI with PFA

First-pass isolation was achieved in all PVs, as reported in a number of previous studies. The reproducibility of these perfect acute results among different centers, operators, and workflows underlines the ease of use and fast learning curve using this ablation system. The real-time feedback on temperature response and impedance tracking may facilitate successful lesion creation. In our small cohort of repeat procedures, PVI durability was high, but the limited patient number does not allow for any conclusive statements to be made. PFA-induced myocardial stunning may explain the recovery of PV conduction in 1 of 7 patients.

### Creation of linear LA lesions

The time required to perform LA linear lesion sets beyond PVI was significantly reduced compared with conventional ablation systems, with an acute efficacy of 100%. Reconduction into the posterior wall after RFA is frequently observed in reablation procedures (75%^23^). Limited delivery of RF energy to preserve the anatomic integrity of the posterior LA wall and protect the esophagus against thermal damage is likely a driver for this. Our initial findings suggest that lesions are more often durable, potentially owing to the relative cardiomyocyte specificity of PFA-based lesions. More remapping data are needed to confirm our results. In patients undergoing invasive remapping, a durable block was also observed for all mitral isthmus lines. This is noteworthy because a durable block of the mitral isthmus line is notoriously difficult to achieve with conventional ablation catheters.^24^, ^25^, ^26^ Our findings are consistent with previous reports on acute efficacy^8^^,^^27^ and early remapping data from index procedures.^9^ However, they represent the first cohort of patients who had undergone multiple previous ablations, providing initial evidence of lesion durability and suggesting that this platform may be a valuable option for challenging and complex atrial arrhythmia ablation procedures.

### Ablation in proximity to the coronary arteries

With PFA, coronary artery spasm has been described with different PFA systems when lesions were created close to the coronary arteries.^28^^,^^29^ This can be mitigated by administering large amounts of nitrates before delivery.^30^ With the lattice-tip catheter, 1 case of severe coronary artery spasm leading to ST-elevation has been described but dedicated studies are lacking.^11^ In our study, PFA was delivered for cavotricuspid isthmus ablation in 6 patients and for posterior mitral isthmus ablation in 23 patients (18 LA endocardially, 5 within the CS). No ST-segment changes occurred, even though nitrates were not administered in this study. More experience is needed to define the safety of the lattice-based system for lesion creation near coronary arteries.

### Study limitations

The current study is an observational, single-center, prospective, nonrandomized analysis. Further studies are needed to support the large-scale reproducibility and assess lesion durability and long-term clinical follow-up in broader patient populations. More remapping data are needed to assess lesion durability and evaluate the optimal ablation workflow for linear ablation lesions. Finally, no coronary angiograms were performed, so subclinical coronary artery spasm could not be evaluated.

## Conclusion

In a large tertiary care center cohort of patients undergoing complex left and right atrial ablation procedures, the novel focal RF/PF lattice-tip catheter enables rapid high-density LA mapping and effective and safe PVI, re-PVI, and creation of additional LA lesion sets.

## Disclosures

Dr My has received a research grant from the European Society of Cardiology. Dr Nies has received a scholarship from the German Research Foundation (Deutsche Forschungsgemeinschaft). Prof Metzner received lecture honoraria and travel support from Medtronic, Biosense Webster, Abbott, Boston Scientific, and LifeTech. Dr Schenker has received educational grants from Johnson & Johnson and travel grants from the German Society of Cardiology. Prof Kirchhof received research support for basic, translational, and clinical research projects from German Research Foundation, European Union, British Heart Foundation, Leducq Foundation, Else Kröner-Fresenius Foundation, Dutch Heart Foundation, the Accelerating Clinical Trials funding stream in Canada, Medical Research Council (United Kingdom), and German Center for Cardiovascular Research, from several drug and device companies active in atrial fibrillation and has received honoraria from several such companies in the past, but not in the last 5 years. P.K. is listed as inventor on 2 issued patents held by the University of Hamburg (Atrial Fibrillation Therapy WO 2015140571, Markers for Atrial Fibrillation WO 2016012783). Dr Rillig is a consultant for Medtronic, KODEX-EPD, Biosense Webster, Boston Scientific, AtriCure, and LifeTech; received travel grants and lecture fees or compensation for advisory board from Medtronic, CardioFocus, Biosense Webster, Abbott, Boehringer Ingelheim, Philips KODEX-EPD, Ablamap, Bayer, Novartis, LifeTech, Boston Scientific, AtriCure, and Lilly; and received research grants from Medtronic.
