# Supplementary material for: Pulmonary vein isolation and beyond: Feasibility and acute outcomes of the lattice-tip dual-energy catheter for complex ablations
Source: Heart Rhythm O2. 2025 Nov 25;7(2):212–20. doi: 10.1016/j.hroo.2025.11.013 (PMC12925793; doi:10.1016/j.hroo.2025.11.013)
Supplement: Supplementary Material [file mmc1.docx]

**Supplementary Material**

**Arrhythmia recurrence during early post-ablation follow-up**

The current follow-up period is limited, and most patients have not yet reached the one-year evaluation defined in our prospective registry. Follow-up data therefore primarily reflect patients who returned to our center for clinical reasons after ablation, introducing potential selection bias and likely overestimating recurrence rates.

Follow-up information was available for 43 of 102 patients.

Of these, 25 patients remained free of arrhythmia recurrence. Seventeen patients experienced recurrence: 13 with atrial fibrillation (AF; 4 occurring during the blanking period), 2 with atrial tachycardia (AT), 1 with premature atrial contractions (PACs), and 1 with supraventricular tachycardia (SVT). Five patients required cardioversion (3 during the blanking period; 4 with persistent AF and 1 with paroxysmal AF). One patient with severe pulmonary fibrosis died from respiratory insufficiency, which was considered unrelated to ablation.

Recurrence rates stratified by arrhythmia type are summarized below:

|  | **Paroxysmal AF**  **(n=15)** | **Pers AF**  **(n=15)** | **Pers AF+AT**  **(n=12)** | **Total** |
| --- | --- | --- | --- | --- |
| **Recurrence** | 5 /15  (33%) | 5/15  (33%) | 7/12  (58%) | 17/42  (40%) |
| **No recurrence** | 10/15  (67%) | 10/15  (67%) | 5/12  (42%) | 25/42  (60%) |

These follow-up data provide an initial estimate of post-ablation outcomes but should be interpreted with caution given the short observation period and potential selection bias.
